# The Information and Communication Technology User Role: Implications for the Work Role and Inter-Role Spillover

**DOI:** 10.3389/fpsyg.2016.02009

**Published:** 2016-12-27

**Authors:** Matthew M. Piszczek, Shaun Pichler, Ofir Turel, Jeffrey Greenhaus

**Affiliations:** ^1^Management & Human Resources, University of Wisconsin–Oshkosh, OshkoshWI, USA; ^2^Management, California State University (CSU), FullertonCA, USA; ^3^Information Systems and Decision Sciences, California State University (CSU), FullertonCA, USA; ^4^Management, Drexel University, PhiladelphiaPA, USA

**Keywords:** identity, roles, spillover, technology, information systems

## Abstract

Management and organization research has traditionally focused on employees’ work role and the interface between their work and family roles. We suggest that persons assume a third role in modern society that is relevant to work and organizations, namely the Information and Communication Technology User (ICTU) role. Based on role theory and boundary theory, we develop propositions about the characteristics of this role, as well as how ICTU role characteristics are related to boundary spanning activity, inter-role spillover with the work role, and work role performance. To this end, we first conceptualize the ICTU role and its associations with work and family roles. We then apply identity theory and boundary management theory to advance our understanding of how the ICTU role is related to criteria that are important to individuals and to organizations, namely self-selection into certain types of work roles and positive and negative inter-role spillover. The implications of this role for theory, research, and practice in management and organizations are discussed.

## Introduction

Information and communication technologies (ICTs) such as smartphones and laptop computers are a part of life in modern society. Many use ICTs for both work and personal purposes (Sproull, 2000). Population surveys indicate that use of personal computers and the Internet at home is increasing (Skinner et al., 2003; File, 2013) and information technology is weaved into daily life from a young age (Willoughby, 2008; Calamaro et al., 2009) and into organizations and organizational processes (Orlikowski and Scott, 2008; Orlikowski, 2010). Many people have access and use ICTs from various places, including home, on the road and work (Xu et al., 2012; Ollier-Malaterre et al., 2013; Tarafdar et al., 2015; Qahri-Saremi and Turel, 2016; Turel, 2016).

Recent research in management and information systems has also focused on the role of ICTs in life in modern society, such as in managing role identity boundaries (Pauleen and Yoong, 2001; Kossek et al., 2006; Golden and Geisler, 2007; Hislop and Axtell, 2011). For instance, Carter and Grover (2015, p. 932) introduced the concept of the information technology identity, defined as “the extent to which an individual views use of an information technology as integral to his or her sense of self,” and have called for more research to understand its relationship with other life identities. Nevertheless, the use of technology has not yet been conceptualized and analyzed as a role in the management and organization literature. This is an important gap to address as conceptualizing information and communication technology user (ICTU) role allows researchers and practitioners to rely on theories and evidence-based knowledge related to individuals’ roles, identities, role salience, and inter-role associations for better understanding how people decide on and interact with ICTs at work and in the personal life domain.

As such, the key over-arching premise of our paper is that many individuals in modern society develop an ICTU role and that acknowledging this role can have important theoretical and practical implications. Specifically, such a conceptualization is essential to understand ICTU role related behavior and how this behavior is related to social structures and processes (Merton, 1957; Katz and Khan, 1978), and may provide a framework for research to better understand the mechanisms through which ICTs impact the way people work and live. ICTs have the potential not only to act as a communication medium, but also to shape social interactions and experiences (Altheide, 1995; Meyrowitz, 1997). The use of ICTs can even create functional (Meshi et al., 2015) and structural (Kanai et al., 2012) changes in people’s brains. ICTs impact individuals’ self-perceptions and self-concept (Orlikowski, 1992), which would suggest that individuals who use ICTs might develop an ICTU role identity based on their interaction with the technology (Reeves and Nass, 1996). Role identities are a key component of a role (Stryker and Burke, 2000).

Understanding role identities is essential to scholarship about roles, role performance, and cross-domain relationships between roles (Greenhaus and Beutell, 1985; Stryker and Burke, 2000). In order to fully understand the effects of the increased prevalence of ICTs on the management of roles, we must explore how individuals identify with and experience ICTs themselves. Recent research focused on the use of ICTs in and out of the work domain shows that the effects of ICT use on individual outcomes such as emotional exhaustion and role conflict vary across individuals (e.g., Derks et al., 2016; Piszczek, 2016). Employees can become stressed when they are threatened by new ICT, when the ICT they have does not meet their needs and desires, and when they cannot access their desired IT (Tarafdar et al., 2015). ICT use is also related to work role performance (Fenner and Renn, 2010) and possibly to collisions of personal and professional identities (Ollier-Malaterre et al., 2013). The ICTU role may help explain variation in the use and experience of ICTs in and out of the workplace.

Drawing on research on the work–family role interface, this article provides first strides toward an integrated treatment of the ICTU role as related to the work role, including positive and negative inter-role spillover with the work role. We first define the ICTU role using role theory and identity theory; we in essence show that the ICTU role exhibits all the characteristics that define a role and we provide preliminary evidence that this role can be salient, at least among some employees. Second, we explain the boundary characteristics of the ICTU role based on concepts from border/boundary management theory. Finally, we integrate these perspectives to develop testable propositions about the ICTU role and its implications for the work role and inter-role spillover. We believe this is important given the paucity of research on how technologies impact boundary management (Golden et al., 2006; Golden and Geisler, 2007; Day et al., 2010; O’Driscoll et al., 2010). Although it would be beyond the scope of the current paper to develop propositions about inter-role spillover with the family role, we see this as an important area for future research.

## The Information and Communication Technology User Role

In the social psychology literature, a role is a mental schema of behavioral expectations people assume within a social system (Katz and Khan, 1978). Put differently and perhaps more simply, a role is defined by a set of prescribed behaviors in a given social situation (Jackson, 1965). Through socialization processes of role sending and compliance, people learn the different roles they may play and the expected behaviors, norms, rights, and duties in each role. Role sending consists of offering information and support for some activities, but not others, influencing an individual’s beliefs of what behavior is appropriate in a role, while compliance consists of behaving in the manner perceived as appropriate to that role as determined through role sending (Katz and Khan, 1978; Chiaburu and Harrison, 2008). All of these become part of a mental schema or cognitive framework for understanding a role and prescribed behaviors (Merton, 1957). Individuals enact and alternate between multiple roles over time (Solomon et al., 1985) and sometimes enact multiple roles simultaneously.

Two important roles discussed in the management literature are work and family roles (Greenhaus and Beutell, 1985). In this article, we argue that many persons, including employees in modern organizations, may develop a third pertinent life role, namely the ICTU role. As we explain later, this role is related in important ways to the work role, and can be enacted in various work situations, in parallel to or disjointedly from the work role. We define the ICTU role as *the requirements, benefits, and behaviors expected of an ICT user in a given social situation*.

That is, persons who use ICTs might assume the ICTU role, and with this they develop an ICTU role identity and are confronted with role expectations that are unique to that role, based on the circumstances of their particular social situation or context at any given time. Some roles, such as that of a sick person (Mechanic and Volkart, 1961) or an ICT user, can be temporary and their expressions will vary according to the situation an individual is in.

Since beliefs regarding ICT-related requirements, benefits and behaviors likely differ from one person to another, people will have an ICTU role which is composed of different components that might be present in their self-schema or identity and may be unique to them. Possible components or manifestations of this role include explicit and implicit attitudes toward ICT, beliefs regarding how fast one needs to respond to messages on their ICT, how psychologically attached they are to their ICT (Turel and Serenko, 2012; Turel et al., 2014), how modern, innovative, “cool” they are with the ICT, how socially e-involved they are, how resilient and self-efficacious they are to new task demands (work or personal) that require the use of ICT, how innovative they are with ICT, what is considered appropriate utilization of the ICT (e.g., is it ok to email a person at midnight? Or make a socially objectionable joke?), can they control the use of ICT and attribution of this control (is it up to them or others), how high is the need for ICT use ranked in their ladder of needs (e.g., is this something they do before eating or instead of having intimate relationships), etc. Note that given the embryonic stage of this conceptualization of the ICTU role, more manifestations and unique facets of this role will likely emerge in the future and the above list is certainly not constant nor is it exhaustive.

These ideas are consistent with key propositions from role theory. Role theory is multidisciplinary and accordingly has multiple conceptualizations (Biddle, 1979, p. 9). Theory and research are clear, however, that a role is defined by role expectations, role identities, and role-related behavior (Biddle, 1979, p. 8). All of these apply to ICT as demonstrated in the next sections. Key propositions from role theory are that: roles are formed when behavior is patterned; individuals are aware of roles and of expectations for these roles (including behavioral norms, expectations of others, etc.); roles persist because of their outcomes or functions (Biddle, 1979). We address each of these aspects of the ICTU role in the sections below. First, we focus on role expectations and role identities. We also consider role boundaries, based largely on boundary theory, which is a more recent advancement in role theory (e.g., Clark, 2000; Golden and Geisler, 2007). Further, we consider the outcomes and functions of the ICTU role as related to the work role and thus inter-role spillover with the work role. We contend that because the ICTU role has these properties, i.e., expectations, identities and role-related outcomes, ICTs can be much more than artifacts that allow one to enact the work role: information and communication technology use, for some, can become a role.

## ICTU Role Expectations

Role expectations are a key aspect of any role (Biddle, 1979). Role expectations are defined as “internalized beliefs and attitudes about (a) the personal relevance of a role, (b) the standards for performance in that role, and (c) the manner in which personal resources (i.e., time, money, and energy) are to be committed to performance of that role (Amatea et al., 1986). There is evidence that many individuals identify as ICT users and are aware of norms and expectations around ICT use in different social situations (Sheldon et al., 2011). Based on key tenets of role theory, this suggests that individuals likely develop an ICTU role. Similar to other roles, there are often general social norms and expectations regarding the ICTU role in and out of organizations (D’Arcy et al., 2009). Through the role sending process, which can occur at different levels of analysis, such as from the organization to the individual or between individuals, these expectations are signaled to ICT users (Anderson et al., 1993). For example, an ICT user must know when ICT use is socially inappropriate, such when conversing with colleagues (Turel and Bechara, 2016). There are norms and expectations regarding specific task–technology combinations such as cell phone use (Judes and Stevens, 2007), Internet use (Whitty and Carr, 2006), and appropriate behaviors in online communities (De Cindio et al., 2003) that apply across nearly all aspects of ICT use. Role expectations are also created by organizations (Lapointe and Rivard, 2005; Barki et al., 2008). ICT use demands and duties are common in many organizations that enforce the use of certain systems (Hartwick and Barki, 1994). For example, hospitals can have technology use norms and expectations such as use of charting software or pagers (Berner et al., 2005; Scott et al., 2005). More generally, many employees experience organizational expectations to use technology to work from home and may suffer workplace consequences when compliance with these role expectations is low (Fenner and Renn, 2010). Research based on role theory has documented role expectations are related to the role stressors, such as inter-role conflict, and to psychological and/or physical strain (e.g., Cooke and Rousseau, 1984). As such, ICT use norms, implicit or implicit, can drive work-family conflict (Turel et al., 2011a) and technology related stress (D’Arcy et al., 2014).

## The ICTU Role and Other Life Roles

**FIGURE 1:**
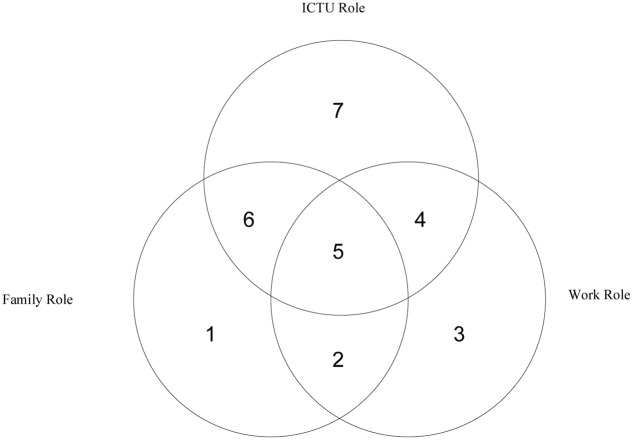
**Life roles in modern society**.

We argue that the management and organization literature can benefit from understanding the ICTU role and its interactions with the work role. While we later focus on the interaction of the ICTU role with the work role, considering the intersection between the ICTU and family roles is informative to our arguments and important for developing an understanding of how ICTU and work roles are related. **Figure 1** depicts an integrated view that amalgamates these three key life roles and visualizes how the ICTU role interacts with work and family roles.

**Figure 1** demonstrates how the ICTU role may overlap with other life roles, and that it has its own life domain as well. The literature on the work-family interface (e.g., Frone et al., 1992; Adams et al., 1996; Heller and Watson, 2005) has focused on area 2. This literature is informative in demonstrating characteristics of the work role boundary and how the work role interacts with other roles. For example, not only can an employee’s mood at work, as affected by the family role, influence job performance, but also job-related phenomena can influence one’s family domain (Carlson et al., 2011). Though it is becoming an increasingly popular area of research, fewer studies have sought to explore the role of technology in this interface; i.e., focused on area 5 (the intersection of work, family, and ICTU roles). Such studies focus mostly on organizational ICT that is used in the family domain (e.g., Golden and Geisler, 2007; Hislop and Axtell, 2011) and rarely on personal technology, such as social media, which is used in the work domain (Ollier-Malaterre et al., 2013) and do not conceptualize technology from a role identity framework. It is important to understand how work pressures are related to ICT use and the ICTU role because employers can require employees to use technology for work purposes off-site (Jacobs and Gerson, 1998). Thus, area 5 potentially encompasses a complex set of triadic influences, which is largely underexplored and beyond the scope of this work.

Areas 4, 6, and 7 have been covered to some extent in the literature. Area 6 represents the intersection of the ICTU role with the family role. These situations can involve physical or psychological interactions with an ICT during family time; or alternatively, family related tasks or thoughts invoked while or instead of physically or psychologically interacting with an ICT. Similarly, area 7 covers the ICT technology user role itself and its behaviors, separate from family and work roles (e.g., while driving, meeting friends, or being away from one’s family) (Bhattacherjee, 2001; Choi and Kim, 2004; Barnett and Coulson, 2010). Persons can still, though, assume this role while at work or with their families or when alone (away from work and their families). It involves the use of ICT for mostly hedonic purposes which are intrinsically rewarding and can require the immersion of the ICTU user in ICT matters, such as solving a technology or installation problem, downloading an app or content which have nothing to do with work or family life (e.g., fitness tracking, games, music, e-books, news) or simply engaging in intrinsically rewarding activities that do not contribute or influence family and work life, such as playing videogames or reading news after all family members have fallen asleep. Assuming this role, for instance, during work time can be, for example, in the form of taking a break from work and using one’s Facebook account to see what his or her friends have been up to.

While areas 6 and 7 (family–ICT intersection, and ICT domains) do not directly relate to the work role, there still may be a spillover from these areas to the working environment. For instance, the use of ICTs in these roles can elicit both positive and negative emotions (Deng and Poole, 2010) as well as influencing sleep patterns (Thomée et al., 2012), which in turn, can influence the work role (Carlson et al., 2011). For example, a person who uses a tablet before going to bed, e.g., just to check the news (area 7) may, by doing so, suppress the release of melatonin, a hormone signaling the body on sleep onset (Wood et al., 2013) and consequently may underperform in the work role due to sleep deprivation (Barnes and Spreitzer, 2015). Such processes are largely understudied, especially among working adults, and merit further research (Tarafdar et al., 2013).

Area 4 covers the interplay between the technology and work domains, which is the focus of this article. The area of work-ICT interaction has been studied quite extensively, but without considering the ICTU role, its characteristics, and its integration with other roles. For example, the fit between ICTs and tasks can influence job performance (Dennis et al., 2008) and ICTs can facilitate online collaboration and virtual team processes and outcomes (Majchrzak et al., 2000; Maznevski and Chudoba, 2000; Colquitt et al., 2002; Martins et al., 2004; Schiller and Mandviwalla, 2007). Research related to the integration of the ICTU and work domains is arguably lacking (Orlikowski and Scott, 2008; Orlikowski, 2010).

## ICTU Role Identity and Identity Salience

Role identity is a composition of meaning that individuals apply to the roles that they occupy (Stryker and Burke, 2000). Role identity is defined as “the meanings people attribute to themselves while in a role” (Stets, 2010). The concept of a role identity is based on a symbolic interactionist perspective rooted in Mead (1934), whose work can be summarized as: society shapes self, which shapes social behavior. In other words, social structures shape how individuals view themselves and how they behave socially (Stryker, 1980). A salient role identity is “conceptualized as being positioned at the top of the [identity] hierarchy” (Callero, 1985, p. 203). In other words, it is defined by how important that identity is to an individual as compared to other identities (Stryker and Serpe, 1982), and has been operationalized as one’s dedication to a role in terms of investing in the role (e.g., Amatea et al., 1986), or “the likelihood that the identity will be invoked in diverse situations” (Hogg et al., 1995, p. 257). In other words, for any given role, for example the work role, individuals will develop an identity or set of meanings associated with that role; that role identity will vary in its salience or importance within the individual vis-a-vis other role identities. For instance, for some individuals their work role identity is more salient than their family role identity (and vice versa for other individuals). In this case, individuals will be more dedicated to the work role, and the work role identity will be more likely to be invoked across different situations. Role identity salience is determined, in part, by the number of relational ties supported by the role and the perceived importance of these ties to the individual (Stryker and Serpe, 1994; Stryker and Burke, 2000).

There is evidence that ICT use can shape an individual’s self-concept, that individuals develop their own sense of how to behave vis-a-vis ICTs and that they develop an identity around the ICTU role (Venkatesh and Morris, 2000; Cyr et al., 2009), all of which would support the over-arching existence of the ICTU role (Biddle, 1979). We acknowledge that not everyone interacts with ICTs, and thus some people may not develop an ICTU role. That said, most adults generally have moderate to extensive exposure to the use of ICTs, at home, work, or both (File, 2013). Notably, those who are familiar with ICTs in society but do not use them still form an ICTU role identity, but they may identify as non-users or incapable users.

Management and information systems research indirectly supports the existence of the ICTU role and consequently of an ICTU role identity (Carter and Grover, 2015). ICT users develop role identities based on their IT role affiliations, e.g., as an organizational user or as a service provider (Gefen and Ridings, 2003), including beliefs about technology skills, opportunities and constraints to use technology, the importance of technology, and motivation to use technology (Goode, 2010). Human-technology interaction research delineates the role of a technology user as an important role (Carter and Grover, 2015) that can become incorporated into users’ daily experiences and actually change perceptions of self-identity and shape interactions with others (Schlosser, 2002). For example, updating one’s Facebook profile can influence one’s impressions of the self (Gonzales and Hancock, 2011) and be driven by social norms, expectations and rules regarding what is acceptable behavior (Lowry et al., 2011). Such ICT use can be fulfilling on its own and be driven by pure intrinsic gains (Davis et al., 1992). Research has also shown that ICT use is related to self-esteem (Gonzales and Hancock, 2011) and to social self-concept (Gil-Or et al., 2015). ICTs can change the way individuals interact with others, the way they respond to stress, and can even affect their brain structures (Kuehn et al., 2011, 2014; Kuehn and Gallinat, 2014). Once a role identity is established, the salience of that role vis-a-vis other life roles becomes defined, and awareness of the subjectively perceived centrality of this role is formed.

The management information systems literature supports variation in ICTU role salience. Individuals vary in the extent to which ICTs are important to them (Limayem et al., 2007), the extent to which they want to interact with ICTs (Compeau and Higgins, 1995), and their interest in engaging with ICTs in new and different ways (Agarwal and Prasad, 1998), all of which would suggest that individuals might attribute varying levels of importance and centrality to their ICTU role identity (e.g., Gefen and Ridings, 2003; Turel et al., 2011a). This literature further implies that interpersonal differences in ICTU identity can be rooted in individual differences in innovativeness (Yi et al., 2006), computer self-efficacy (Marakas et al., 2007), beliefs and perceptions regarding technologies (Venkatesh, 2000), social pressures to use technologies (Titah and Barki, 2009), impression management (Turel et al., 2010), and adherence to ICT related policies (Warkentin et al., 2011).

Generally, we do not expect the ICTU role to be more salient than major life roles such as work and family. However, in extreme cases a person may place the ICTU role high in the role salience hierarchy (Turel et al., 2011b) and at times become cognitively absorbed in ICT use (Saade and Bahli, 2005; Lin, 2009) to the point that this role overrides other life roles in terms of salience (Charlton and Danforth, 2007). This can lead to addiction-like symptoms among ICT users (American Psychiatric Association, 2013) which means that in some cases the ICTU role may dominate other life roles more fully (Kuss et al., 2013). Recent neuroscience evidence suggests that the use of ICTs can reinforce incentive rewards, making the ICTU role central to one’s identity, i.e., a higher ICTU identity salience (Meshi et al., 2013).

## ICTU Role Boundaries

As part of the sociocognitive role formation process, individuals form mental role boundaries between roles in the mind. Role boundaries cognitively constructed heuristic perimeters around a role that determine their scope, typically in terms of time or space (Ashforth et al., 2000). These boundaries vary in terms of flexibility and permeability. The ability of ICTs to be used as tools to enact roles across domains (e.g., answering work e-mails while at home) is related to its fairly unique role boundary permeability and flexibility. The permeability of a role boundary is the extent to which the boundary allows thoughts, behaviors, attitudes, or emotions associated with another role to enter (e.g., Ashforth et al., 2000; Clark, 2000). Boundary flexibility is the degree to which a role can be enacted outside of typical or prescribed spatial and temporal boundaries of the role (Ashforth et al., 2000; Clark, 2000). The ICTU role is highly flexible and permeable and therefore can be engaged in many locations and at any time because of the mobility of ICTs. Because of this, enactment of the ICTU role may overlap in time and space with the enactment of other roles. For example, checking one’s social media accounts at work may engage the ICTU role in the physical and temporal space of the work domain. This rapid transition between roles can lead to the blurring of the ICTU role with other roles and to inter-role influences (Turel et al., 2011a).

The ICTU role boundary is flexible and permeable in relation to the work role because it regularly expands to accommodate work-related behavior. For instance, if an individual receives a work-related call while commuting on a train to work, the ICTU role becomes activated, but the individual has some choice in terms of whether to take the call or not. This is important, in part, because it implies that individuals can choose, with varying degrees of freedom, depending on certain circumstances and constraints, the extent to which ICTs are utilized across situations, including at work. This choice can be to holistically adapt ICT use beyond its original intent, e.g., from the social domain to the work domain (Ashforth et al., 2014). As with other roles, engagement of materials or tools that are related to a particular role is unnecessary for a role identity to be activated. For instance, one does not need to be caring for a child for the family role to be activated, nor does ICT use need to occur for the ICTU role to be active. Some ICT users report anxiety called “fear of missing out” when ICTs are not readily accessible (Przybylski et al., 2013). That said, the use of materials and tools related to a role can, of course, activate that role psychologically and behaviorally and the permeability and flexibility of ICT boundaries make this particularly likely for the ICTU role.

Another important factor relating to role boundaries is the desired levels of role segmentation or integration with other roles. According to boundary theory and related research (Nippert-Eng, 1996; Ashforth et al., 2000) individuals vary in their general preference to integrate or segment roles, i.e., to simultaneously or disparately enact roles, respectively. Roles that are permeable and flexible are easier to integrate because their boundaries expand to encompass behaviors associated with other roles. The high degrees of role boundary flexibility and permeability may make the ICTU role seem relatively nebulous as compared to other roles, namely work and family roles, especially because ICTs can be used to enact these roles (e.g., to call a family member). That said, cognitive sociological theory argues that individuals form role boundaries around people, places, things, and ideas by “lumping” together those that occur together and “splitting” those that do not (Zerubavel, 1993). If ICT use appears across multiple domains, individuals will be less likely to associate ICTs with another life domain and instead form boundaries around the ICTU role itself. Consistent with arguments above, the more domains ICT use appears in, the more social ties it generates and the less likely a person will lump ICTs into another role domain. As a result, as ICT use becomes more ubiquitous in society individuals will be more likely to form a discrete ICTU role. We argue that, into the future as ICT use becomes more prevalent, individuals will, in general, be more likely to recognize the ICTU role as a distinct and important life role.

**FIGURE 2:**
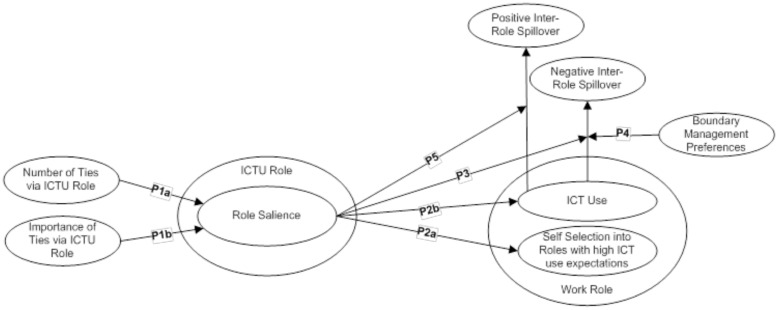
**Conceptual model of ICT and work role interactions**.

Now that we have established the concept of, and potential value of, the notion of an ICTU role, its attributes and its interface with other life roles studied in the management and organization literature, we further leverage theory to develop the concept of this role in terms of its implications for work role-related behavior. We develop propositions about the ICTU role and its characteristics; role boundaries and the ICTU role; and inter-role spillover between the ICTU role and the work role. These propositions are illustrated in **Figure 2**.

## Implications for Inter-Role Spillover with Work

Now that we have established the concept and potential value of the ICTU role, we further leverage concepts explained above from identity theory, boundary theory, and spillover theory to develop the concept of the ICTU role in terms of its implications for work role-related behavior. We develop propositions about the ICTU role and its characteristics; role boundaries and the ICTU role; and inter-role spillover between the ICTU role and the work role. These propositions are illustrated in **Figure 1**.

### Role Characteristics and ICTU Role Behavior

#### Role Identity Salience

As noted above, role identity salience is related to the felt importance of a particular role identity, as well as involvement and commitment in a role (Amatea et al., 1986). Different identities vary in their salience within individuals. The salience of each is hierarchically ordered within individuals. Some individuals will feel that the use of ICTs is important to them to the extent it represents an important aspect of who they are and be more committed to the ICTU role and role-related behaviors, such as ICT use.

When an identity is more salient, it is more likely to be considered, diffused into and enacted across situations and social interactions (Stryker, 1968; Burke and Reitzes, 1981). Consequently, more social ties or stronger social ties across other roles facilitated by ICT use should be associated with a greater distinction between the ICTU role and other life roles, thus increasing its salience in the mind of the individual (Utz, 2010). Identities are more salient when sacrificing the identity means sacrificing social relationships, especially those that are important to the individual (Stryker, 1968). In other words, salience is higher when the number or the intensity of dependent relationships is relatively high for a given identity. Identity salience is important because it predicts the likelihood that an individual will behave in a way that is consistent with that identity (Wiley, 1991). The larger the number of persons an individual perceives he or she is connected to because of his or her ICTU role, or the more important those individuals are to the individual, the more salient the role identity will be.

Proposition 1: (a) The number of one’s network ties via the ICTU role is positively related to one’s ICTU role salience, and (b) the importance of these network ties to the individual is positively related to ICTU role salience.

### Role Boundary Characteristics and the ICTU Role

Because a key characteristic of the ICTU role is its highly flexible and permeable boundaries, there can be significant individual variation in its enactment across other role domains. For example, individuals who prefer role integration may be more willing to perform work outside the typical work role domain using ICTs (Ollier-Malaterre et al., 2013). Role boundary flexibility and permeability are associated with role integration and blurred role boundaries and facilitate the enactment of the ICTU and work roles that allow or require ICT use (Ashforth et al., 2000).

Role identity salience may help explain when and why individuals engage in optional ICTU role enactment. When a role identity is more salient, role-relevant behavior is more likely (Wiley, 1991); individuals seek to act in ways that confirm their role identities (Stryker, 1980). Because work roles can often facilitate ICT use, we propose that role salience is an important predictor of the extent to which individuals attempt to find overlap or consistencies between the ICTU role and work role. This proposition is based on the person-job fit literature, which suggests that individuals are attracted to jobs that expect behavior consistent with their individual characteristics and preferences (Edwards, 1991). As such, individuals with a relatively salient ICTU identity should be more likely to search for and engage in work roles consistent with their ICTU identity. For instance, an individual with a relatively high ICTU role salience will be more likely to seek work that requires the use of ICTs for work-related purposes. This is consistent with research on role theory, which has shown that a more salient role identity for a given role is related to more activity in that role as compared to other roles (e.g., Greenhaus and Powell, 2003). This is also consistent with research on applicant attraction and job choice, which has consistently shown that perceived fit with the work role is related to attraction and, hence, to job choice (e.g., Chapman et al., 2005). That said, we would suggest that role identity salience is only one among perhaps many factors in self-selecting into work roles with varying levels of ICT use and to ICT use in the work role.

Occupying a work role with high ICT use expectations is not necessary to enact the ICTU role. Individuals with salient ICTU role identities are also more likely to use ICTs as a substantive means of communication, even in a work role that does not require it or that facilitates other means of communication (Guo et al., 2010). In other words, those with high ICTU role salience generally choose to use ICTs to facilitate work when possible, even when other circumstances do not allow them to self-select into a work role that requires heavy ICT use. For example, some students are more inclined than others to engage in ICT-mediated learning (Liang et al., 2011) and employees may innovate with ICT to avoid manual work (Hopkins and Brynjolfsson, 2010). Although this is, in part, a function of certain individual differences, preferences and experiences (see Fenner and Renn, 2004, 2010), we suggest that this is also a function of ICTU role salience.

Proposition 2: Information and Communication Technology User role salience is positively related to (a) self-selecting into work roles with high ICT use expectations, as well as (b) using ICTs in the work role.

### Inter-Role Spillover between the ICTU Role and the Work Role

To develop propositions about spillover between the ICTU and work roles, we draw upon research on spillover between the work and family roles. Research on work and family roles and their intersection has been influenced greatly by a spillover perspective (Geurts and Demerouti, 2002), which suggests that what happens in one role has implications for and can affect what happens in another. The literature has focused on two types of spillover: negative spillover and positive spillover. The literature has focused largely on the former and has therefor defined inter-role spillover as “a within-person, across-domains transition of strain from one area of life to another” (Bakker and Demerouti, 2013). That said, spillover can be either negative or positive, and thus involve spillover of not only conflict but also enrichment. Negative spillover—or inter-role conflict—between roles, is defined as “pressures arising from one role are incompatible with pressures arising in another role” (Greenhaus and Beutell, 1985). Inter-role conflict occurs between two roles when meeting the demands of one role makes it more difficult to meet the demands of the other role (Greenhaus and Beutell, 1985). Positive spillover—also known as enrichment—between roles is defined as “the extent to which experiences in one role improve the quality of life in the other role” (Greenhaus and Powell, 2006).

A seminal paper by Greenhaus and Beutell (1985) proposed three types of inter-role conflict or negative spillover (specific to work and family): behavior-, time-, and strain-based, each of which has been studied and supported in the literature. Behavior-based conflict occurs when behaviors expected in one role are inconsistent with behaviors expected in a different role. Time-based inter-role conflict occurs when time required or spent in one role detracts from time necessary to perform another role. Strain-based inter-role conflict occurs when one role causes psychological strain, which spills over into another role, affecting the latter role in an adverse way (e.g., role satisfaction or performance). This perspective has occupied much of the literature on work-family conflict and meta-analyses of work-family conflict tend to leverage this perspective (e.g., Michel et al., 2010).

We expect that inter-role spillover also occurs between the ICTU and work roles. While we expect this influence to be bidirectional, we focus on spillover from the ICTU role to the work role because it demonstrates how the ICTU role can have important implications for the workplace. We identify several factors that affect how employees experience their ICTU and work roles and the intersections between these roles in terms of both positive and negative spillover: the degree to which the work role and the ICTU role are inconsistent, an individual’s preference to integrate the ICTU role into the work role, and ICTU role salience. We propose that individuals can experience both positive and negative spillover between the ICTU role and the work role depending on the level of consistency and inconsistency in these roles, respectively. That is, when work role expectations around the use of ICTs are consistent with one’s preferred level of use based on their ICTU role identity salience, positive spillover is more likely to occur. When work role expectations are inconsistent with one’s preferred level of use based on their ICTU role identity salience, then negative spillover is more likely. We also expect that boundary management strategies are important such that there are differences between segmenters and integrators when it comes to how inconsistencies between roles are related to negative inter-role spillover. These broad propositions are developed and explained in more detail below in terms of more specific, testable propositions.

#### Negative Inter-Role Spillover

We argue above that those with highly salient ICTU role identities will seek to enact this role by seeking work that allows ICT use and will be more likely to use ICTs for work. But what happens if, for instance, such a person ends up in a work role that does not facilitate the enactment of ICTU role behaviors? The degree to which the ICTU role can be integrated into the work role will depend on the match between an individual’s ICTU identity salience and work role expectations. Both Ashforth et al. (2000) and Clark (2000) propose that the similarity between roles is related to spillover between them. For some individuals, the ICTU role and the work role may be very similar and easily integrated. This is the case, for instance, when one’s ICTU role is highly salient and the work role requires a high degree of ICT use. For others, the roles may be very dissimilar, requiring different types of behaviors, and are thus less easily integrated. This is likely the case for individuals whose ICTU identity salience is relatively low but whose work role requires extensive use of ICTs. If an individual’s ICTU role identity is relatively more (less) salient, then the proscribed (prescribed) use of technology for work purposes may cause behavior- and strain-based conflict between the ICTU role and the work role. Behavior-based conflict may occur because expected ICT use behavior in the work role is inconsistent with the ICTU role. Strain-based conflict may occur because the individual is relatively unable to behave in a way that is consistent with a role that is important to the individual, in this case the work role.

For instance, an individual with low ICTU role salience attributes little importance to the ICTU role, does not view the role as a central part of the self-concept, and may prefer not to communicate via ICTs. If an individual with low ICTU role salience is in a job with high ICT use expectations (e.g., to communicate via instant messaging) he or she is likely to experience behavior- and strain-based conflict between the two roles because the behaviors expected by the work role are inconsistent with the individual’s ICTU role identity. Relative to individuals who have a salient ICTU identity, this will tend to cause stress for this worker because use of information and communication technologies is uncomfortable. This is consistent with the work-family literature: role demands are more strongly related to negative spillover or inter-role conflict when role identity salience is higher (Greenhaus and Beutell, 1985). The greater the differences between one’s work role expectations and ICTU identity salience, the more likely the individual will experience behavior- and strain based inter-role conflict. In other words:

Proposition 3: Information and communication technology use in the work role is positively related to negative spillover between the ICTU role and work role for persons with a less salient ICTU role identity, and is negatively related to negative spillover between the ICTU role and work role for persons with a more salient ICTU role identity.

Even if a person’s ICTU role salience and work role are consistent with each other, a person might not desire to integrate them. As Clark (2000) asserts, there is no ideal state in terms of segmentation vs. integration of roles: the effect on the individual depends on his or her desire for segmentation or integration, and well-being can be achieved through either approach. Research has shown that a consistency between an individual’s preference for segmentation or integration of work and family roles, for instance, and actual segmentation or integration is related to higher job and family satisfaction and less depression and stress (e.g., higher job and family satisfaction, lower depression and stress, Edwards and Rothbard, 1999; Kreiner et al., 2009).

Likewise, we expect that a consistency (inconsistency) between an individual’s preferred boundary management strategy and one’s expected or prescribed level of work role ICT use is related to relatively lower (higher) inter-role conflict (holding salience constant). ICTs can be used in a variety of ways, i.e., mostly for work, mostly for non-work, or in a more integrated fashion (Golden and Geisler, 2007), and individuals vary according to their preferences in this sense. It is important to understand the extent to which an individual’s work role is consistent with his or her preferences for boundary management with the ICTU role. Individuals who prefer to segment (integrate) ICTU and work roles will find a particular work role more stressful when it prescribes a high (low) level of the use of ICTs. Consistency between work role requirements in terms of ICT use and an individual’s preference for segmentation or integration of the ICTU and work roles will therefore be negatively related to behavior-based inter-role conflict.

Proposition 4: Information and communication technology use in the work role is positively related to behavior-based negative spillover between the ICTU role and the work role for those who prefer to segment the ICTU role from the work role, and is negatively related to behavior-based negative spillover between the ICTU role and the work role for those who prefer to integrate the ICTU role with the work role.

#### Positive Inter-Role Spillover

In contrast to a resource scarcity perspective, which suggests that negative spillover occurs because different roles compete for the same resources, a role accumulation perspective suggests that occupying multiple roles is related to an increase in rewards and resources (Sieber, 1974). As with other roles, the ICTU role can be related to certain rewards (e.g., self-esteem), resources (e.g., valuable skills), and demands (e.g., attending to electronic notifications). What is perhaps particularly interesting about the ICTU role is that resources allocated to this role, such as time, do not necessarily detract from resources devoted to other life roles, such as work, as is usually found with work and family roles because individuals regularly engage with technology to get tasks done in other roles, such as the work role (Dennis et al., 2008). In other words, due to its flexible and permeable boundaries, the ICTU role can potentially facilitate the enactment of other roles.

If identity salience is related to investment in a role, then ICTU role salience should be related to motivation around use of ICTs, which can potentially facilitate the completion of certain work tasks. This would suggest that successful task performance via ICTs may be influenced by the way in which the person engages psychologically with the technology as a relatively more (or less) important aspect of self, that is, their ICTU role identity. Whereas dissatisfaction with one role may normally cause persons to seek satisfaction from other life roles (Champoux, 1978), satisfaction with the ICTU role may be related to increased satisfaction with the work role. We accordingly expect that positive spillover, e.g., positive emotions or moods, will occur when engaged in work roles that can be facilitated by the use of ICTs for individuals with a relatively salient ICTU identity. Whereas before we argued that *inconsistency* between roles creates *negative spillover*, here we argue that *consistency* between roles creates *positive spillover*.

Proposition 5: The relationship between ICT use in the work role and positive inter-role spillover between the ICTU role and the work role is positive for persons whose ICTU role identity is relatively high in salience and negative for persons whose ICTU role identity is relatively low in salience.

## Discussion

Information and communication technologies have the power to diminish boundaries between persons and groups of persons, as well as between physical and social environments (Meyrowitz, 1997). Cerulo (1997) contends that the ability to reproduce and share internal thoughts and experiences with others via ICTs changes the human experience and social interactions, and that more social-scientific research is needed to understand the impact of technology on the self, behavior, and social interaction. This was a major purpose of this paper: to understand the impact of technology not only on behavior such as the use of ICTs in the work role, but also on identity itself and how it relates to both psychological (i.e., inter-role conflict) and behavioral (i.e., self-selection into work roles) outcomes. Role behavior is sometimes difficult to predict, in part, because the self is complex and differentiated based on multiple role identities (Stets and Harrod, 2004; Riketta and Nienaber, 2007) and because technology can contribute to a more differentiated sense of self (Evans, 1995) and help in enacting other roles. Individuals in modern society occupy a number of roles which, of course, makes for an interesting if not complicated self and way of life, perhaps especially for working persons facing multiple and varied demands, sometimes conflicting, from multiple life roles (Williams et al., 1991).

### Implications for Theory and Research

#### The ICTU Role

An overarching assertion developed in this article is that individuals often occupy a role of ICT user (ICTU) to some degree, which can become an important aspect of the self with the potential to impact, both positively and negatively, other life roles, and particularly the work role (Ahuja et al., 2007; Innstrand et al., 2008). The ICTU role identity varies in salience across individuals. Unfortunately, the ICTU role has not yet received significant attention in the management and organization literature, although it has been implied that people develop role identities related to their ICT use preferences and affiliations. Identifying and investigating this role is important, we argue, because doing so has implications for our understanding of the work role, inter-role spillover, and ultimately work role performance. It also allows researchers to conceptualize and study the interaction between the use of ICTs and work related outcomes from a role theory perspective. This is valuable because role theory is useful for understanding organizational behaviors and processes (Reichers, 1985; Eagly et al., 1995; Singh, 2000), and we argue, also the way individuals interact with technologies at work.

#### ICTU Role Identity and Identity Salience

This article developed the concept of an ICTU role identity and leveraged identity theory (Stryker and Burke, 2000) and boundary theory (Nippert-Eng, 1996; Ashforth et al., 2000) to explain the development of the role identity and its likely role characteristics. We argued that individuals develop an identity as an ICT user based not only on ICT use, but on interactions with others while engaged in the ICTU role as well as the reactions of others, their expectations for role behavior, and the individual’s internalization of these reactions and expectations. The ICTU role identity becomes more salient due to social circumstances as well: the number and strength of one’s network ties associated with the ICTU role will increase the salience of the role. Today, this likely happens over the life course as individuals are bombarded with ICTs from a young age (Sproull, 2000; Calamaro et al., 2009). These propositions suggest that one’s social context helps to shape one’s role identity salience, which is consistent with the broader literature on role identities (Wiley, 1991; Zerubavel, 1993, 1996), which we hope has laid the groundwork for scholars to further understand the ways in which one’s social environment might shape the ICTU role and role related behavior. For instance, it might be helpful to understand how coworker role expectations about ICTs are related to individuals’ use of ICTs in the work role and how this is influenced by one’s ICTU role identity salience. It would also be useful to study if enactment of the ICTU role identity could trigger enactment of other life roles, such as the work role.

It is important to understand the salience of the ICTU role, in part, because of its implications for the work role. Based on an integration of perspectives from identity theory and boundary theory, we proposed that ICTU role salience is related to self-selection into work roles that involve use of ICTs, as well as the use of ICTs in the work role more generally. While there is no direct evidence in support of this proposition, as the ICTU role is a new concept, it is reasonable to expect so based on the stress (Lazarus and Folkman, 1984) and technology-related stress (Tarafdar et al., 2015) literatures. Both streams suggest, for example, that when a person has too many ICT demands which presumably do not match their ICTU role salience (stressors), they will cope with such stressors through various maladaptive coping strategies such as avoidance (not selecting a job), disengagement (avoiding ICT use on the job) or more adaptive strategies, such as adjusting ICTU role salience, for instance through taking ICT courses. ICTU role salience can affect primary appraisals of ICT stressors (e.g., new ICT may be more stressful for employees with low ICTU role salience) as well as secondary appraisals relating to one’s ability to cope with the stressors (e.g., the perceived ability may be lower for employees with low ICTU role salience). Recent research also finds that ICTs can be a job resource and thus help reduce stress when it grants employees control over their role boundaries (Piszczek, 2016). This research also suggests that the ability of employees to draw boundary control from ICTs depends on individual characteristics such as ICTU role salience. This also suggests that for some individuals, the inability to use ICTs (e.g., if one’s smartphone breaks or the organization adopts a policy prohibiting after-work e-mails) may be particularly difficult. As such, the magnitude of stress reactions resulting from the ICTU role and its management likely vary substantially across individuals.

These appraisals, as influenced by ICTU role salience, can lead to employment choices as a means to cope with the ICT-related stressors in one’s environment. While ICTU role salience may not always elicit an extreme reaction such as self-selection into a job, is likely to have many practical organizations implications. An individual with low ICTU role salience may, for example, be less responsive to e-mail communications in general if they prefer to communicate in person. Such individuals may not respond to work communications at all outside of the workplace because they are not likely to be engaged in ICT use. Such proposition illustrates the potential relevance of ICT role salience, though, validating them requires further research.

The functional and adaptive uses of ICTs in work roles are increasingly important across a variety of jobs and occupations (Nolan and McFarlan, 2005; Harris et al., 2013; Raviola and Norback, 2013). Thus it is important to be able to understand the characteristics of individuals that predict such use beyond more general attitudes toward technology; ICTU role salience is such a factor that can be assessed by employers. This has implications for the literatures on person-environment fit, personnel selection, and performance management. We believe it will be increasingly important for management and organization scholars to understand and leverage the features of the ICTU role when developing theory about the ways in which individuals are best suited to their jobs, how performance in rapidly changing work roles can be most effectively managed, and how technology can be most effectively integrated into various work roles.

#### Boundary Management

We further proposed that boundary management strategies explain the relationship between work-role ICT use and negative spillover between the ICTU and work roles. We know that individuals vary in their preferences for engaging with ICTs in general and in the work role (e.g., Turel and Serenko, 2012). Differences in such preferences may be related to differences in attitudes towards different types of roles. We suggest a key to understanding how effective ICTs are across different persons in work roles is their ICTU identity and how their ICTU identity might be expressed at work based on boundary management preferences. To be sure, even if a particular individual has a salient ICTU role identity, this same individual may want to keep this part of the ICTU role relatively separate or segmented from work. We feel this is an important contribution, in part, because of the lack of research on how technologies are related to boundary management (Golden and Geisler, 2007). This would be exemplified by two persons who, for instance, are connected to many of their friends and family through technology, who have relatively salient ICTU identities and consider themselves avid information and communication technology users, and who may even be so “techy” that they help friends and family with technology problems. Yet, one individual prefers to keep this identity separate from the work role (e.g., not talking about this part of oneself much) and the other prefers to fully integrate this with the work role (e.g., bringing this up at work and trying to excel through his or her ICT expertise).

#### Inter-Role Spillover

Information and Communication Technology User role experiences—expectations, demands and so on—can crossover to other life roles in both negative and positive ways. Based on a role accumulation perspective (Sieber, 1974) and consistent with research on work-family enrichment (Greenhaus and Powell, 2006), we proposed that work role ICT use and ICTU role salience are relevant simultaneously as to positive inter-role spillover such that consistency between work role ICT use and ICTU role salience is related to positive spillover between roles. In this sense, experiences in the ICTU role can enrich experiences in the work role. If persons’ ICTU role is a central aspect of their identity, they will be more likely to have positive experiences with ICTs in the work role, e.g., they might be quicker and/or more successful in adapting to the implementation of new information technologies in a given work role. This is an important conceptual integration of propositions from the literatures on identity and positive inter-role spillover, and we hope that future research will consider how consistency between individuals’ work roles and their ICTU identities is related to positive implications for the work role, such as job performance.

#### Organizational Context

Individuals seek to behave in a way that is consistent with their role expectations and the norms of the group within which they are embedded (Swann et al., 1992). Cerulo et al. (1992) suggest that communication technologies are relevant for collective identities, in the sense that they can create a sense of community or unity among persons. Organizational context can be an important higher-level determinant of employee attitudes, behaviors, and outcomes (Oldham and Cummings, 1996; Somers, 2001; Korsgaard et al., 2002). It can also be a source for self-identity formation (Hogg and Terry, 2000) through self-verification processes (Swann et al., 1992). We therefore expect that when organizational contexts involve and support ICT use, self-verification of ICTU roles will be enhanced, and employees will behave more consistently with the ICTU role as a means to self-verify their ICTU and work identities. This is important because improper use of ICTs in organizations is growing (D’Arcy and Hovav, 2009; Tarafdar et al., 2015), and this problem can be tackled through architecting and reinforcing desired ICTU roles through organizational contexts. Thus, organizational context can serve as a boundary condition for forming, maintaining, and re-shaping ICTU identities. One key area for future research is the function of organizational context in the development and enactment of the ICTU role.

Overall, more research, conceptual and empirical, should be conducted to better understand how the ICTU role is related to the work role. Such studies can also inform related research on work and family roles. Further research should examine the nature of the ICTU role itself as well as the potentially complex, yet important, set of interactions among family, ICTU, and work roles. Given the growing prevalence, and presumed salience of the ICT role (Turel and Serenko, 2010), such research can help us better understand and find means to improve the delicate balance many employees maintain nowadays, among work, family and ICT use.

### Implications for Practice

The propositions point to several interesting avenues of managerial action. For example, ICTU role identity seems to be an important driver of conflict with the work role. Thus, under permissible regulations, it can be assessed in recruitment processes, and used as a basis for employee placement. Alternatively, it can be leveraged for increasing employee productivity by letting employees shape their ICT user role (e.g., by participating in technology implementation projects). A second example stems from the proposition that individuals with similarly salient ICTU and work role identities who are presented with a situation where there is no clear behavioral response consistent with only one role, may experience stress, which in turn will impair their performance. To mitigate such situations, appropriate behavioral responses should be developed, and employees could be provided with training regarding how to react with the appropriate behavioral responses in such situations.

Boundary management preferences can be assessed during recruitment or employee development processes, and be used for hiring and placement decisions. When the provided boundaries do not match employees’ expectations or preferences, employees’ performance is likely to diminish. Lastly, organizational contexts can be re-shaped (e.g., by providing technical training and support) so as to try to impact individuals’ ICTU identities. It could also be used (e.g., by means of flexible IT use policy) to improve the fit between employees desired and actual ICTU-to-work segmentation (or integration). The merits of such measures, though, require further research.

## Conclusion

The role theory perspective we advance is important given the paucity of research taking into account how complex socio-technical systems are related to role performance (Orlikowski, 1992, 2010; Avgerou and McGrath, 2007; Orlikowski and Scott, 2008). Role and boundary management theories have been developed to explain how identities and role demands are related to important role-related behavior, such as job performance (Katz and Khan, 1978; Solomon et al., 1985; Kossek et al., 1999, 2006; Golden and Geisler, 2007; Gal et al., 2008). By applying these relatively well-developed theories to a relatively new, yet pertinent, role of ICTU, scholars can better understand the complex relationships between technology use and behavior in the ICTU and work roles. Although previous research has focused on how technology per se is related to job performance (Gallupe et al., 1994; Straus and McGrath, 1994), our contribution is more specific to how role identities surrounding technology, not technology per se, are related to role behavior.

## Author Contributions

MP and OT came up with the original idea for the ICTU role and thus the manuscript in consultation with JG. SP lead the literature review in coordination with the other three co-authors. MP lead on revisions of the manuscript after presentations at the Academy of Management and Work-Family Researchers Network, and JG helped edit the manuscript as well and focus the paper in terms of contributions to theory and research.

## Conflict of Interest Statement

The authors declare that the research was conducted in the absence of any commercial or financial relationships that could be construed as a potential conflict of interest.
